# Enhancing the comparability of costing methods: cross-country variability in the prices of non-traded inputs to health programmes

**DOI:** 10.1186/1478-7547-4-8

**Published:** 2006-04-24

**Authors:** Benjamin Johns, Taghreed Adam, David B Evans

**Affiliations:** 1 Health System Financing (EIP/HSF), World Health Organization, 9^th ^Floor Bina Mulia – I Building, Jl Rasuna Said Kav. 10, Kuningan Jakarta 12950, Indonesia; 2 Health Systems Financing (EIP/HSF), World Health Organization, Geneva, Switzerland

## Abstract

**Background:**

National and international policy makers have been increasing their focus on developing strategies to enable poor countries achieve the millennium development goals. This requires information on the costs of different types of health interventions and the resources needed to scale them up, either singly or in combinations. Cost data also guides decisions about the most appropriate mix of interventions in different settings, in view of the increasing, but still limited, resources available to improve health.

Many cost and cost-effectiveness studies include only the costs incurred at the point of delivery to beneficiaries, omitting those incurred at other levels of the system such as administration, media, training and overall management. The few studies that have measured them directly suggest that they can sometimes account for a substantial proportion of total costs, so that their omission can result in biased estimates of the resources needed to run a programme or the relative cost-effectiveness of different choices. However, prices of different inputs used in the production of health interventions can vary substantially within a country. Basing cost estimates on a single price observation runs the risk that the results are based on an outlier observation rather than the typical costs of the input.

**Methods:**

We first explore the determinants of the observed variation in the prices of selected "non-traded" intermediate inputs to health programmes – printed matter and media advertising, and water and electricity – accounting for variation within and across countries. We then use the estimated relationship to impute average prices for countries where limited data are available with uncertainty intervals.

**Results:**

Prices vary across countries with GDP per capita and a number of determinants of supply and demand. Media and printing were inelastic with respect to GDP per capita, with a positive correlation, while the utilities had a surprisingly negative relationship. All equations had relatively good fits with the data.

**Conclusion:**

While the preferred option is to derive costs from a random sample of prices in each setting, this option is often not available to analysts. In this case, we suggest that the approach described in this paper could represent a better option than basing policy recommendations on results that are built on the basis of a single, or a few, price observations.

## Introduction

Cost and cost-effectiveness studies are increasingly being used to guide practical policy decisions in many parts of the world. This tendency has received additional impetus in low income countries with international attention focused on how best to achieve the health-related millennium development goals (MDGs). The numerous resulting studies explore the impact of different mixes of interventions on health as well as the resource requirements to scale them up to the extent necessary to achieve the goals [1-4].

Costing and cost-effectiveness studies have, however, frequently included only the costs incurred at the point of delivery to beneficiaries, omitting those incurred at other levels of the system. These additional costs have been defined elsewhere as programme costs and we use that terminology in this paper [5]. They include the costs of establishing and running a programme at national, provincial or district administrative levels, for activities such as planning and supervision, advertising and media, and training.

In some published studies it is not clear whether programme costs have been included. Where they are included it has been common to assume that they are an arbitrary proportion of total costs – commonly 10% – although at other times programme costs estimated from other countries have been converted to the local setting at official or purchasing power parity (PPP) exchange rates [6]. The few studies that have attempted to estimate programme costs directly have shown that they vary by intervention and can range from a relatively low to a substantial proportion of total costs [7,6]. The total exclusion of programme costs, or inaccurate estimation methods, can misrepresents the resources required to implement or maintain health interventions and biases comparative assessment of their cost-effectiveness.

In its CHOICE project [9], WHO seeks to assist countries to set priorities by making publicly available region- and country-specific estimates of the costs, effectiveness, and cost-effectiveness of a large set of interventions, analysed in a standardized and comparable way [10]. The project was developed in response to the recognition by countries that it is not feasible for each of them, particularly those with limited capacity, to perform full analyses of the cost-effectiveness of all possible ways of spending scarce health resources in their settings. Subsequently, WHO-CHOICE has also provided assistance to global and country efforts to estimate the resources required to scale up health interventions, including those aimed at achieving the millennium development goals [11]. In this work, WHO has sought to include programme costs as accurately as possible and in a standardized way, in addition to the costs incurred in providing services directly to patients – e.g. for pharmaceuticals, investigatory tests, outpatient visits and hospital stays.

An ingredients approach to costing has been used where the physical quantities of the necessary inputs are multiplied by their unit prices to obtain total costs. To facilitate this work, data have been collected on the unit costs or prices of the different types of inputs and activities used in key interventions for as many countries as possible. This has revealed that unit costs vary considerably both within and across countries. For this reason, the common practice in cost-effectiveness studies of basing cost estimates on a single observation of the cost of an input – for example, the price of labour, transportation or renting an office – can lead to misleading results [6]. This has already been illustrated by Adam et al. [10] for hospitals where the cost per inpatient day was shown to vary by an order of magnitude across hospitals within the same country. If an analyst were to use the unit cost estimated for a hospital that happens to be an outlier, high or low, the results would be biased either against or towards hospital-based interventions. Moreover, it would not be possible to know the direction or extent of any possible bias.

For this reason, we have tried to explore alternative ways of obtaining average unit costs for cost-effectiveness analysis aimed particularly at countries where there are limited data points available from accurate costing studies. Clearly the first and best solution would be to base costs on a representative sample of observations for all possible inputs and activities, but rarely do analysts have access to these data or the funds to generate them, even in the richest countries.

Results for inpatient days and outpatient visits to hospitals have already been published [10] and those for department-specific hospital stays and labour costs are under review [10,12-14]. This paper describes models focusing on two "non-traded" inputs to programme costs – media and advertising for health promotion, and utilities such as water and electricity that are essential ingredients of most health programmes. Media can be a very small or a very large component of costs, at the limit approaching 100% of costs for mass media campaigns for health promotion, for example. Utilities, on the other hand, are almost always present but rarely constitute a large part of costs.

There are three objectives. The first, and overall, objective is to develop a method that can be used to estimate the average cost of an input for countries with limited data points. The two non-traded inputs that are the focus of this paper are used to illustrate the method, not because they are the most important non-traded inputs for all interventions.

The remaining two objectives are specific to these two inputs. The second objective is to understand the determinants of variations in the unit costs of these inputs across and within countries. The third is to use the estimated relationships to impute an average cost for each country, taking into account the observed variation, uncertainty, and the levels of the other determinants. It is postulated that this average cost is likely to be a more appropriate unit price to use in an economic analysis than a single observation that might well be an outlier.

The paper has, therefore, two audiences. The first consists of analysts who conduct or interpret costing and cost-effectiveness studies. The second comprises researchers and policy makers seeking to understand the methods that have been used in the WHO-CHOICE project.

The paper is organized as follows. For each of the two inputs (media and utilities) the methods and results are presented in turn, including the imputed prices using the best-fit model for a selected number of countries. We conclude with an overall discussion of the potential uses of these results for policy.

## Methods and results

### Theoretical considerations

Where a good is traded internationally and no producer or consumer is sufficiently large to influence price, the purchase price (free on board or *fob*) available to all importing countries should be the same [15]. Differences in unit costs observed across countries would, under these circumstances, be attributable to differences in transport, insurance and internal distribution costs. Many inputs to health are traded internationally and the markets are approximately competitive – examples are generic pharmaceuticals and items of medical equipment where there are many alternative suppliers. In this case there would be a single internationally competitive price which would reflect the marginal cost of production and the marginal value to the international consumer of the last unit purchased.

On the other hand, the market for brand name pharmaceuticals is not competitive with different purchasers being able to negotiate different prices with the monopoly producers [15]. There could be numerous international prices for inputs such as these, determined partly by a country's capacity to pay and its bargaining power. Price will certainly be above the marginal costs of production [16] and variations in prices across countries would be expected to be positively correlated with GDP per capita or some other measure of ability to pay.

Where goods are not traded internationally, the interaction of domestic supply and demand influences price. Assuming that no producer or consumer is sufficiently large to influence price and that the government does not intervene, price would be the point where the domestic supply and demand curves intersect, or where the marginal valuation of benefit by consumers equals the marginal cost of production. When the good is an input to production, such as cement for building, or paper for printing, competitive markets would imply that price is equal to the marginal cost of production (which determines the supply curve) as well as the value of the marginal product produced (which determines the demand curve).

The demand curve would shift outwards with increases in GDP per capita because of the income effect. Given that wages for most types of labour are positively correlated with GDP per capita, [17] the supply curve would shift upwards as GDP per capita increases – e.g. each unit of output would cost more in richer countries. Taken together, and assuming away, for the moment, other influences on prices such as differences in production technologies, the price of pure non-traded goods would be positively correlated with GDP per capita.

The interaction of supply and demand described above assumes that production technology does not change across countries with increases in GDP per capita. If, for example, there are economies of scale and the long run average cost curve continues to fall with increases in output, countries with larger markets could be operating on different short run average and marginal cost curves associated with different scales of production [18]. Countries with smaller markets would be seen to have supply curves that are above those of countries with larger markets, and thus have higher prices than larger markets. Accordingly, although a positive relationship would still be expected between the price of pure non-traded goods and GDP per capita on average, the relationship might not be found if significant economies of scale in production existed – something that is possible with utilities in particular.

The relationship will also be mitigated by many other factors that can influence supply and demand. For example, the extent of any foreign direct investment [19], the degree of international trade in the products that use that input or factor of production [20], the extent of union power [21], and the nature of market structures [22] have been identified as possible determinants of price or wage variations across countries. Input prices will also be influenced by variations in the strength of government regulation or involvement in the market. Depending on the relative influence of these factors, variation in prices across countries would be more strongly or weakly correlated with variations in GDP per capita.

The two "non-traded" inputs under discussion here are really intermediate factors of production. They are inputs to the production of health, but in turn require physical inputs such as labour, equipment, and raw materials. Some of these are traded internationally, while others are not (and thus, the goods under discussion are not 'pure' non-traded goods). Market structures differ as well. Media outlets and utilities are sometimes owned by government, with little or no competition. In other cases the producing companies are privately owned and operated, usually with some market power to affect prices. Their ability to do so depends on the extent and nature of government regulation and control.

While we would still expect that prices will vary across countries with national ability to pay and variation in the costs of production, both positively correlated with national income or GDP per capita where markets are approximately competitive, the strength of the relationship would still be moderated by differences in domestic market conditions across countries and the extent to which the two non-traded intermediate inputs themselves use traded factors of production. At the extreme, however, if governments effectively set prices for utilities or for media outlets, it is not clear what type of relationship would be observed.

Accordingly, we hypothesize here that a positive relationship would be expected between GDP per capita and unit price except in the case of pure traded goods where prices should not differ very much across settings. However, we recognize that the strength of this relationship would depend on a number of factors influencing supply and demand including the extent to which our intermediate inputs use traded primary factors of production and the extent of market regulation or control by government. At the extreme, if governments control prices directly, it is possible that a negative relationship, or no relationship, would be observed depending on the principles driving their pricing policies.

### Data collection

In the course of 2000–2001, the WHO-CHOICE project hired 19 consultants from different countries chosen to span different epidemiological and income regions of the world. A workshop demonstrated a standardized cost data collection tool (CostIt) and a detailed list of ingredients or inputs to the various activities required to develop or maintain a programme or intervention. This was designed to increase the comparability of data-collection methods across interventions and countries, and to reduce measurement error (for more information see [7]).

In addition to the data collected by the consultants, data from a variety of additional sources were identified and added to the data set. These are described separately for the two categories of non-traded inputs subsequently. All of the data were examined for outliers. When outliers were detected, the observation was checked to ensure that it had been entered accurately. In a few cases, outliers lacking a plausible explanation were eliminated. The data were entered into STATA software, which was also used for the analysis [23].

The following sections describe the data, model specification and results for the two programme cost inputs in turn. Tests for goodness of fit, retransformation methods, estimation of predicted values and uncertainty intervals are only presented after the first model as they are the same for all models.

### 1. Flyers, posters, and advertising

#### Data

Various forms of information dissemination are critical to health promotion but are also used to provide information to populations about the availability of curative or preventive activities such as immunization days. It can be divided into two broad categories: printed materials for distribution, and the use of advertising time or space in media outlets such as radio, television and newspapers. For the former, informational flyers and posters were taken as representative of the broader category. To maintain cross-country comparability of prices, flyers were defined as one A4 or comparable sheet of paper printed on both sides, and posters were defined as one square metre. The costs of media advertising were based on one minute of television, one minute of radio (both during daytime but not prime time), and a quarter page advertisement in the body (i.e. not the front or last page) of a newspaper.

While media companies often provide "free" advertising to public health initiatives, this is not the case in every country, and in any case these activities use scarce time or space that could be used for other purposes. Accordingly, the costs should be included in the analysis where a societal perspective is taken [5]. Since advertising charges vary by the size of the audience reached, they were collected at national, provincial, and district levels, together with estimates of the number of people potentially reached. In addition to the data on prices collected by the consultants, data on advertising rates were gathered from the Internet by searching the web-sites of printing companies, radio and television stations and newspapers. Languages used in these searches were English, French, Spanish, Russian, Vietnamese, Portuguese and Arabic. The internet searches identified 214 observations from 66 countries. The prices were deflated or inflated to year 2000, the base case for the analysis, by applying consumer price indices (CPI) from central-bank or similar government agency web-sites, or CPIs from international organizations when the central bank did not report them.

#### Model specification

Separate regressions were run for printed material and advertising in the mass media. The dependent variable in both is unit cost as a percentage of GDP per capita. Using a percentage allows costs to be analysed in a metric easily converted into all currency units. The first explanatory variable is GDP per capita as described above, which was tested in terms of both international dollars (denoted by the symbol I$ – converted from domestic currency to I$ using purchasing power parities) and US dollars (US$ – converted at official exchange rates). (For prediction purposes, it makes no difference whether the dependent variable is the ratio of price to GDP or simply the price itself – mathematically, the elasticity of price to GDP per capita is simply our beta coefficient plus unity e.g. 1+β. This is discussed further in the results section.)

A variety of regional dummies were tested to see if there were region-specific characteristics that might influence prices. We hypothesized that countries that were geographically contiguous were more likely to have interrelated markets and similar price structures, so tested the following regional dummies: sub-Saharan Africa, Latin America, Europe, Eastern Mediterranean/Middle-east, Asia, Oceania and North America. At different times the dummies for Asia were further subdivided into "South Asia" and "Eastern Asia" and Europe was divided into Eastern and Western Europe. As an alternative, we also tested grouping countries into those that were developed and less developed, and explored other income classificatory systems such as that utilized by the World Bank.

For media advertising, we had more than one data point from a number of countries. To correct for the possible interdependence of the error terms because of this clustering, the regressions were re-run using the robust (cluster) option in STATA, based on the Huber-White method of robust variance estimation [23]. In this case, the coefficients are identical to those in OLS regressions, but the variance, and therefore the significance, of the coefficients can differ.

Third, a number of factors reflecting the domestic supply and demand for advertising time and space were considered. These included the size of the population in the service area which is likely to make advertising more attractive and raise demand, and therefore prices [24]. A variant of this, the predicted market size (*MS*), which takes into account the number of people with access to the particular form of media, was also tested [25,26]. It is calculated as:

*MS*_*i *_= *PS*_i _* *PA*_*i*_,     (Equation 1)

where *PS*_*i *_is the population of the area served by the media outlet in the area where the *i*th observation is found, and *PA*_*i *_is the percentage of the population with access to the media outlet. For television and radio, we used the proportion of people with television or radio[27] as a proxy for access, and the adult literacy rate was used for newspapers [28].

On the other hand, the extent of competition between media outlets would likely reduce prices, so the extent of competition in the market was also tested as an explanatory variable. Competition was tried first as the number of outlets per potential market audience. This assumes that newspapers, televisions, and radio outlets do not compete with one another. Because there is some evidence that print, radio, and television advertising is substitutable, the extent of competition between all forms of media was also tested [28]. Dummy variables indicating monopoly of the market and whether the government owned or operated the media outlet (where unit costs could be higher or lower than private sector outlets) were also tested as factors influencing the nature of the interaction between supply and demand. Table 1 summarizes the explanatory variables used at various times in each of the models.

**Table 1:** Independent variables explored for each model

**Model**	**Variable Name [Source/Justification]**	**Variable Description**	**Source**	**Expected Influence *ceteris paribus***
All	GDP (PPP) [16]	Gross Domestic Product measured in international dollars	WHO	Prices increases with GDP
	GDP (USD) [16]	Gross Domestic Product measured in US dollars	WHO	Prices increases with GDP
Both Media Models	Regional Dummies (WHO)	Global Burden of Disease regions (geographic and economic)	WHO	Proximate groups may have similar market structures
	Regional Dummies (WB)	World Bank regions – Income groups (economic)	WB	Proximate groups may have similar market structures
Printed Media	Flyer Dummy	Variable indicating if price is for flyer rather than poster	Collected data/WHO	Lower price if flyer
	Flyer Dummy * GDP	Interaction term		Price of flyers may have different relation to GDP than posters if different printing technologies are used
Advertising Media	Population in service area [17]	Total population in area reached by a media outlet	UN Stats/World Gazetteer	Larger population raises price
	Predicted market size [17]	Total population in area reached by a media outlet adjusted for access to media outlets	UN Stats	Larger population raises price
	Competition within media outlet type [23;24;25]	Number of media outlets within a category (TV, Radio, Newspaper), adjusted and unadjusted for predicted market size	CIA Fact book/UN Stats	Greater competition would likely reduce prices (although interacts with demand for media)
	Competition with all media outlet types [23]	Number of media outlets across categories (TV, Radio, Newspaper), adjusted and unadjusted for predicted market size		
	Monopoly [17]		Collected data	Monopoly would likely raise prices
	Government Ownership [17]			Undetermined; depends on government pricing policy but likely would raise prices if monopoly or lower prices if in competitive market.
	Newspaper Dummy	Variable indicating if price is for Newspaper rather than radio	Collected data/WHO	Higher price than radio
	TV Dummy	Variable indicating if price is for TV rather than radio		Higher price than radio
	Dummies * GDP	Interaction term		Price of media may have different relation to GDP than radio because different technologies are used
Water	Access to fresh water	Amount of water available per capita (annual)	WB Development Indicators	Higher access to water should lower price
	Total quantity of water supplied [17;33]	Total amount of water supplied in a country (annual)		More demand should raise prices; may also indicate dis/economies of scale
	Island (dummy)	Variable indicates if the country is a small island	Data collected	Increase price if an island
	Annual Rainfall [33]	Total annual rainfall	Country Watch	Increased rainfall should decrease price of water
Electricity	Fraction derived from fossil fuels	Percentage of electricity generated from fossil fuels	CIA Fact book	Higher fossil fuel use should increases price
	Fraction imported	Percentage of electricity consumed that is imported		Higher imports should increase price
	Total electricity consumption [17]	Total amount of electricity consumed		More demand should raise prices; may also indicate dis/economies of scale
	Total electricity production [17]	Total amount of electricity produced		

**Table 2:** Smearing factor for each regression model

Model number	Input explored	Smearing factor
1a	Printed media	1.693
1b	Advertising media	2.334
2a	Water	1.249
2b	Electricity	1.193

**Table 3:** Results of regression for printed materials

Number of observations = 35				
	Adjusted r^2 ^= 0.5446	F statistic = 21.33	P of F statistic < 0.0001	
Variable	Coefficient	SE	T	P

ln GDP per capita	-.9194	0.1835	-5.01	0.000
Flyer cost (dummy)	-1.558	0.3767	-4.13	0.000
Constant	-.2874	1.623	-0.18	0.861

Dependent variable: log ratio of price of printed materials to GDP per capita

Breusch-Pagan test of heteroskedasticity: 1.02 (p = 0.31 (Chi2)).

VIF test for multicolinearity:1.02 (less than 2 indicates no multicollinearity)

**Table 4:** Results of regression for advertising time or space

Number of observations = 214				
Adjusted r^2 ^= 0.6116		F statistic = 56.90	P of F statistic < 0.0001	
Variable	Coefficient	SE (Robust)*	T (Robust)*	P (Robust)*
ln GDP per capita	-0.592	0.0967 (0.1310)	-6012 (-4.52)	0.000 (0.000)
ln of population in service area	0. 425	0.0461 (0.0482)	9.20 (8.81)	0.000 (0.000)
Dummy if costs are for TV	2.215	0.2559 (0.2705)	8.66 (8.19)	0.000 (0.000)
Dummy if costs are for newspaper	2.055	0.2286 (0.2093)	8.99 (9.82)	0.000 (0.000)
Dummy for Eastern Europe	-8.668	4.1304 (4.1141)	-2.10 (-2.11)	0.037 (0.039)
Dummy for Eastern Europe * ln GDP per capita	1.0670	0.4688 (0.4673)	2.28 (2.28)	0.024 (0.026)
Constant	-5.1640	1.1805 (1.4728)	-4.37 (-3.51)	0.000 (0.001)

*The results as reported after a robust and country clustering are reported in the parenthesis.

Dependent variable: log ratio of price of media and advertising to GDP per capita

Breusch-Pagan test of heteroskedasticity: 1.91 (p = 0.1667 (Chi2)).

VIF test for multicollinearity is not appropriate for this model due to the inclusion of interaction variables.

Ordinary least squares (OLS) regression was used in the analysis. After exploring the model variables for non-linearity and experimenting with possible transformations, the natural log transformation was used for both the dependent and the explanatory variables that showed evidence of non-linearity, since this form best approximated a normal distribution of the variables. It is shown in Equation 2:



where ln denotes natural logarithms, UC_*i *_is the unit cost (poster, flyer, or advertisement) in the *ith *country in year 2000 and GDP_*i *_is the GDP per capita of the ith country in 2000. In all equations, X_1 _is the natural log of GDP per capita in 2000 of the country where the *i*th observations is located and *e *denotes the error term. Regional dummies were included as explanatory (X) variables in all equations as were possible interactions between these dummies and GDP per capita.

For posters and flyers, the only additional explanatory variable included in the equation was a dummy variable indicating that the observation was for a flyer rather than a poster. The effect of interacting this variable with GDP per capita was also explored.

For advertising time and space, in addition to the variables described above, a dummy variable was included for observations relating to newspapers, and another for those relating to television advertising. In interpreting the coefficients, the base case was, therefore, radio advertising.

#### Goodness of fit

Standard regression diagnostic procedures were used to check all models, including those in subsequent sections, for misspecification and goodness of fit. These procedures included exploration of the correlation between independent variables; estimation of the tolerance test and its reciprocal variance inflation factors (VIF) for multicollinearity; visual inspection of residual plots and statistical assessment for non-linearity and heteroskedasticity; and the Breusch-Pagan test for heteroskedasticity [23].

#### Imputing average prices and uncertainty analysis

Once the equations explaining variation in unit costs had been estimated, two types of uncertainty need to be considered when they are to be used to predict prices for the different settings. Uncertainty in the α and β coefficients of equation 2 derives from having the fact that the equation is estimated from a sample of observations from the entire possible data set; fundamental uncertainty occurs because the independent variables do not explain all of the variation in the dependent variable [29]. To account for these types of uncertainty, statistical simulation following the logic of survey sampling is used to impute average unit costs, standard deviations, and confidence intervals. The methods used to determine predicted values and uncertainty ranges have been described in detail elsewhere [10] and are summarized briefly here.

Two steps are involved. First, simulated parameter values are obtained by drawing random values from the normal distribution of the estimated parameters using their variance co-variance matrix to obtain a new value of the parameter estimate. This is repeated 1000 times. Second, simulated predicted values of ŷ (the quantity of interest) are calculated, as follows: (1) one value is set for each explanatory variable; (2) taking the simulated coefficients from the previous step, the systematic component (g) of the statistical model is estimated, where g = f (X, B), this results in 1000 values of g; (3) the predicted value "ŷ" is simulated by taking a random draw from the systematic component of the statistical model, this is repeated 1000 times to produce 1000 predicted values, thus approximating the entire probability distribution of ŷ. From these simulations, the mean predicted value, standard deviation, and 95% confidence interval around the predicted values are computed. In this way, this analysis accounts for both fundamental and parameter uncertainty.

Since the predicted values are estimated in natural logarithms, simply taking the anti-log of these values to retransform them to natural units results in a biased estimate because it provides the median, not the mean, values. This bias is due to the model variables having a normal distribution in log scale, which is not the case in natural units. Duan 1983 presented a solution to this bias, called the smearing factor, and is applied here [30]. This is done by multiplying the antilog of the estimated values by the smearing factor, which is the mean of the antilog of the model residuals. Separate smearing factors were calculated for each regression equation, as reported in Table.

## Results

Table 3 shows the result of the best fit model for posters and flyers. The only statistically significant variables are GDP per capita and the dummy for flyers (versus posters). The fit of the model was better using GDP per capita in international dollars rather than in US dollars using official exchange rates. This was true for all subsequent models. The adjusted R squared is 0.54, with an F statistic of 21.33 (p < 0.0001), indicating that the model explains more than half of the variation of the cost of printed media across countries.

The coefficient for GDP per capita is negative and its absolute value is just less than one (-0.92, p < 0.0001). This is the elasticity of the dependent variable with respect to a change in GDP per capita, suggesting that a 1% increase in GDP per capita results in a fall in the ratio of the unit cost of posters and flyers to GDP per capita of 0.92%. The elasticity of unit price (as opposed to the ratio) with respect to GDP per capita is 1+β, as shown earlier, or 0.09 in this case. Unit costs of printed material are, therefore, inelastic with respect to GDP per capita – they rise as GDP per capita rises, but at a much lower rate than GDP per capita. The dummy variable indicating whether the price is for a poster or flyer (flyer = 1) has a negative coefficient and indicates that the price of a flyer is approximately 21% of the price of a poster (p < 0.0001). None of the interactions proved to be significant.

Table 4 presents the best fit results for media advertising using the robust cluster option. The independent variables explain a greater share of the variation in the price ratio than they do for posters and flyers (R squared = 0.61) and the F statistic is high and significant (F = 56.9; p < 0.0001). There are no problems with multicollinearity or heteroskedasticity, and all variables remain significant after controlling for clustering.

The relationship of unit cost to GDP per capita is more complex than for the printed materials because of the interaction variables, and the elasticises (of price, not the ratio) derived from the equation are summarized in Table 5.

**Table 5:** Elasticises of the unit price of advertisements with respect to GDP per capita

	Eastern Europe	Rest of the World
Media	1.475	0.408

All prices increase with GDP per capita. Unit prices are lower than would be expected for the level of national income in Eastern Europe (the coefficient for the regional dummy is negative and significant), but prices rise with GDP/capita more rapidly than in the rest of the world. In fact, price is elastic in Eastern Europe and inelastic in the rest of the world.

Prices are also positively correlated with the size of the population in the service area, in this case probably reflecting the fact that advertisers are willing to pay more if they reach more people rather than indicating diseconomies of scale in covering a wider population. The variables for media outlets per potential audience were significant only when size of the population served is not included in the equation; the two variables also showed relatively high correlation (0.63). Since the size of the population served was found to be more significant, only this variable was retained in the equation. None of the other supply or demand variables proved to be significant in the best equation.

### Imputing average prices per country

Table 6 and Table 7 report the imputed average prices for each country based on the reported equations and the methods described earlier, in year 2000 International (I$) and US dollars (US$). Standard deviations and 95% confidence intervals for each estimate are also provided. Full details for all countries and regions are available at [9].

**Table 6:** Estimated price for print media for selected countries in 2000 I$ and US$

Country	GDP per capita (I$)	In or out-of-sample^1^	Type	Price per unit^2,3^				Price per unit	
				
				Mean (I$)	95% uncertainty interval Low	95% uncertainty interval High	SD	Mean (US$)	SD
Mali	636	Out	Flyer	0.51	0.21	1.01	0.27	0.16	0.09
			Poster	2.42	0.95	4.63	1.25	0.77	0.40
Mozambique	720	Out	Flyer	0.51	0.22	0.99	0.25	0.15	0.07
			Poster	2.42	0.99	4.59	1.20	0.72	0.36
Indonesia	3,168	Out	Flyer	0.54	0.33	0.83	0.15	0.13	0.03
			Poster	2.55	1.51	3.95	0.76	0.59	0.18
Ecuador	3,261	In	Flyer	0.54	0.33	0.82	0.15	0.18	0.05
			Poster	2.56	1.51	3.96	0.76	0.86	0.25
Algeria	3,949	Out	Flyer	0.55	0.35	0.81	0.15	0.25	0.07
			Poster	2.59	1.55	3.97	0.74	1.17	0.33
Romania	6,412	Out	Flyer	0.56	0.36	0.81	0.14	0.14	0.04
			Poster	2.69	1.64	4.04	0.75	0.68	0.19
Russian	8,036	In	Flyer	0.58	0.37	0.84	0.15	0.13	0.03
Federation			Poster	2.74	1.67	4.15	0.78	0.62	0.18
Bahrain	14,159	Out	Flyer	0.61	0.35	0.95	0.18	0.48	0.14
			Poster	2.91	1.62	4.65	0.95	2.30	0.75
Greece	16,706	Out	Flyer	0.62	0.35	0.98	0.19	0.39	0.12
			Poster	2.96	1.60	4.81	1.02	1.85	0.64
United Arab	20,331	Out	Flyer	0.63	0.34	1.04	0.21	0.64	0.21
Emirates			Poster	3.03	1.56	5.09	1.12	3.06	1.13
United	24,348	In	Flyer	0.61	0.35	0.95	0.18	0.60	0.18
Kingdom			Poster	2.91	1.62	4.67	0.96	2.87	0.95
Canada	28,088	In	Flyer	0.66	0.33	1.13	0.25	0.54	0.20
			Poster	3.16	1.47	5.61	1.31	2.57	1.06

^1^In and out of sample indicate whether or not the country was included in the data set used to estimate the model.

^2^Regional estimates are available at .

^3^Unit price is for a flyer of size A4 double sided, and a poster of a size of one meter square.

**Table 7:** Estimated prices for advertising media for selected countries in 2000 I$ and US$

Country	GDP per capita (I$)	In or out-of-sample^1^	Type of advertising media	Price per unit of advertising (national average)^2^				Price per unit	
				
				Mean (I$)	95% uncertainty interval Low	95% uncertainty interval High	SD	Mean (US$)	SD
Mali	636	Out	Television	1772.03	1105.46	2668.48	489.50	560.31	154.78
		Out	Newspaper	1489.14	923.03	2264.68	411.30	470.86	130.05
		Out	Radio	190.79	121.38	275.33	49.07	60.33	15.52
Mozambique	720	Out	Television	2277.33	1438.27	3354.40	604.92	676.68	179.74
		Out	Newspaper	1913.69	1204.12	2858.08	507.10	568.63	150.68
		Out	Radio	245.66	158.49	353.13	62.27	72.99	18.50
Indonesia	3,168	Out	Television	11752.53	7883.70	16583.24	2809.93	2720.73	650.50
		Out	Newspaper	9843.86	6678.58	13627.51	2195.09	2278.87	508.17
		Out	Radio	1281.47	794.18	1901.86	344.83	296.67	79.83
Ecuador	3,261	In	Television	3556.87	2545.69	4866.86	718.19	1192.26	240.74
		In	Newspaper	2970.61	2218.11	3871.12	493.90	995.75	165.55
		In	Radio	383.67	277.29	510.29	70.12	128.61	23.50
Algeria	3,949	Out	Television	5579.44	3940.72	7621.37	1141.47	2520.54	515.67
		In	Newspaper	4660.45	3534.15	6064.28	793.14	2105.38	358.30
		Out	Radio	603.97	420.44	826.24	123.28	272.84	55.69
Romania	6,412	Out	Television	11931.15	7562.81	17810.30	3064.35	3027.21	777.50
		In	Newspaper	10031.18	6478.71	14708.77	2596.96	2545.15	658.91
		In	Radio	1290.93	838.86	1884.05	336.05	327.54	85.26
Russian	8,036	In	Television	37487.68	21956.51	58201.85	11622.29	8491.69	2632.68
Federation		In	Newspaper	31502.10	18313.14	50166.71	9695.72	7135.85	2196.27
		In	Radio	4082.98	2321.96	6608.19	1380.80	924.88	312.78
Bahrain	14,159	Out	Television	1843.03	1158.26	2683.85	471.42	1455.81	372.37
		Out	Newspaper	1526.35	1098.16	2025.85	283.85	1205.66	224.21
		Out	Radio	196.87	140.40	263.72	38.15	155.50	30.14
Greece	16,706	Out	Television	12714.93	8149.36	18321.48	3183.83	7932.25	1986.24
		Out	Newspaper	10577.81	7488.06	14608.00	2159.53	6599.01	1347.23
		Out	Radio	1381.84	841.69	2034.56	368.24	862.07	229.73
United Arab	20,331	Out	Television	6485.48	4215.24	9211.70	1552.37	6542.02	1565.90
Emirates		Out	Newspaper	5382.99	4008.62	7129.34	955.36	5429.92	963.69
		Out	Radio	700.30	461.54	991.37	159.36	706.40	160.75
United	24,348	Out	Television	3879.47	2430.51	5551.92	979.47	3820.69	964.63
Kingdom		In	Newspaper	3213.12	2337.67	4222.01	586.00	3164.43	577.12
		In	Radio	416.75	283.01	583.22	90.70	410.44	89.33
Canada	28,088	In	Television	12740.92	7628.18	18964.36	3576.09	10346.65	2904.07
		In	Newspaper	10566.88	7213.28	14966.46	2390.91	8581.15	1941.61
		In	Radio	1382.21	810.45	2108.17	399.06	1122.47	324.07

^1^In and out of sample indicate whether or not the country was included in the data set used to estimate the model.

^2^Regional estimates are available at .

### 2. Utilities

#### Data

The three main categories of utilities used in the production of health services across countries are electricity, water, and telephone-based communications. The price of electricity was standardised at the cost of one kilowatt-hour (KWh) and for water, at one cubic metre. The sources of data for electricity included, in addition to the 19 consultants, the United States Department of Energy, the World Energy Organization and the Governments of South Africa, Namibia, and Botswana for their respective countries [31,32]. This resulted in a data set on prices from 60 countries. The WHO and UNICEF Joint Monitoring Programme for Water Supply and Sanitation undertook an extensive survey of the cost of water for a large number of countries, resulting, together with the consultant data, in a data set from 86 countries for water.

The International Telecommunications Union publishes the costs of local telephone calls for all but a handful of countries [33]. The published rates are often standardised at the national level, so we simply took these data for WHO-CHOICE rather than attempting to develop our own models. Telecommunication costs are not, therefore, discussed further in this paper.

#### Model specification

#### Water

The OLS regression specification is the same as Equation 2 above, where the dependent variable is the ratio the unit cost of a cubic metre of water in the country covered by the *ith observation*, in year 2000 prices, divided by GDP per capita in that year. X_1 _is again GDP/capita, as in all the models. To reflect demand [34], access to fresh water and the total quantity supplied were tested as explanatory variables, although the latter might also indicate the existence of economies or diseconomies of scale on the supply side [35]. Two purely supply side variables were explored [34] – a dummy variable indicating if the country is a small island, and annual rainfall [36]. Small islands have more rapid run-off rainfall and less capacity to store water than other settings. All continuous variables were incorporated in natural logarithms as described earlier.

#### Electricity

A body of literature exists explaining variation in the price of electricity on daily or spot markets, where markets have been deregulated [37-41]. However, short term fluctuations are more volatile than longer run changes, although this literature helps identify possible explanatory variables [42-44]. Following equation 2, X_1 _is again GDP/capita. The percentage of electricity generated from fossil fuels in the country [45] and the fraction of total consumption derived from imported electricity were used to reflect supply conditions. In the absence of economics or diseconomies of scale, the ratio of price to GDP is expected to be higher in countries with a greater demand, where fossil fuels are used and where electricity is imported. To reflect demand, total electricity consumption and production were explored separately [45], although these variables might also reflect the existence of economies of scale.

## Results

Table 8 shows the results of the best fit regression model for the price ratio of water. The equation fits the data well with an adjusted R squared of 0.79, with an F statistic of 160.79 (p < 0.0001). The coefficient for the quantity of water supplied is not significant in the best fit model. As described earlier, because the dependent variable is a ratio, the elasticity of price with respect to the quantity of water supplied is 1+β (the β is the coefficient of the independent variable in equation 2). A β of -0.0592 implies an elasticity of 0.9408 – price rises virtually proportionally to the increase in quantity consumed. This suggests either that there are diseconomies of scale, or that the size of the population demanding services allows prices to be increased. On the other hand, price falls slightly with rises in GDP/capita across countries – the elasticity of price with respect to GDP/capita is -0.124.

**Table 8:** Results of regression for price of water in m^3^

Number of observations = 86	Adjusted r^2 ^= 0.7899	F statistic = 161	p of F statistic < 0.0001	
Variable	Coefficient	SE	T	P
ln GDP per capita	-1.124	.0645	-17.43	0.000
ln Total Water Consumption	-.0592	.0460	-1.29	0.201
Constant	1.457	.6044	2.41	0.018

Dependent variable: log ratio of price of water in m3 to GDP per capita.

Breusch-Pagan test of heteroskedasticity:1.87 (p Chi2 = 0.17)

VIF test for multicolinearity:1.03 (less than 2 indicates no multicollinearity)

The best fit regression model for electricity is presented in Table 9. Again, the model fits the data well as judged by the adjusted R squared (0.79) and the F statistic (76.02; p < 0.0001). Unit prices increase with an increase in demand (proxied by consumption) with an elasticity of 0.89 (or 1+ the β coefficient of -0.1098), and with an increase in the percentage of electricity generated from fossil fuel. The elasticity here is almost 2 (1.893) suggesting that price increases almost twice as rapidly as the increase in the percentage of fossil fuel electricity supplied. Hydropower, nuclear power, or other means of electricity production appear to be lower cost ways of generating electricity than by using fossil fuels.

**Table 9:** Results of regression model 2b for price of electricity in KWh

Number of observations = 60	Adjusted r^2 ^= 0.7923	F statistic = 76.02	p of F statistic < 0.0001	
Variable	Coefficient	SE	T	P
ln GDP per capita	-1.090	.0941	-11.58	0.000
ln total electricity consumption	-.1098	.0410	-2.68	0.010
ln percentage of electricity generated from fossil fuel	.107	.0544	1.97	0.054
Constant	.1366	.7399	0.18	0.854

Dependent variable: log ratio of price of electricity in KWh to GDP per capita.

Breusch-Pagan test of heteroskedasticity: 2.55 (p Chi2 = 0.11)

VIF test for multicolinearity:1.20 (less than 2 indicates no multicollinearity)

Price is inelastic with respect to GDP per capita but again the relationship is slightly negative (1 - 1.09 = -0.09), so that countries with higher levels of national income produce electricity at slightly lower unit prices than poorer countries.

### Imputing average prices of water and electricity per country

Table 10 reports the estimated values, standard deviations, and 95% confidence intervals for the unit prices of water per cubic meter and electricity per KWh for a selected number of countries.

**Table 10:** Estimated price of water and electricity for selected countries in 2000 I$ and US$

Country	GDP per Capita (I$)	In or out-of-sample^1^	Type	Price per unit^2^				Price per unit	
				
				Mean (I$)	95% uncertainty interval Low	95% uncertainty interval High	SD	Mean (US$)	SD
Mali	636	In	Water (m^3^)	1.42	1.15	1.72	0.18	0.45	0.06
		Out	Electricity (kWh)	0.37	0.25	0.52	0.09	0.12	0.03
Mozambique	720	In	Water (m^3^)	1.37	1.12	1.65	0.17	0.41	0.05
		Out	Electricity (kWh)	0.26	0.17	0.38	0.07	0.08	0.02
Indonesia	3,168	In	Water (m^3^)	1.13	0.99	1.28	0.09	0.26	0.02
		In	Electricity (kWh)	0.19	0.15	0.24	0.03	0.04	0.01
Ecuador	3,261	In	Water (m^3^)	1.07	0.89	1.25	0.11	0.36	0.04
		In	Electricity (kWh)	0.21	0.17	0.25	0.02	0.07	0.01
Algeria	3,949	In	Water (m^3^)	1.35	1.04	1.69	0.20	0.61	0.09
		Out	Electricity (kWh)	0.22	0.18	0.26	0.02	0.10	0.01
Romania	6,412	Out	Water (m^3^)	1.18	0.97	1.41	0.14	0.30	0.04
		Out	Electricity (kWh)	0.18	0.16	0.21	0.02	0.05	0.01
Russian	8,036	Out	Water (m^3^)	0.96	0.79	1.15	0.11	0.22	0.02
Federation		Out	Electricity (kWh)	0.13	0.10	0.17	0.02	0.03	0.00
Bahrain	14,159	Out	Water (m^3^)	1.24	0.83	1.75	0.28	0.98	0.22
		Out	Electricity (kWh)	0.23	0.18	0.28	0.03	0.18	0.02
Greece	16,706	Out	Water (m^3^)	0.97	0.76	1.19	0.13	0.61	0.08
		In	Electricity (kWh)	0.18	0.14	0.21	0.02	0.11	0.01
United Arab	20,331	Out	Water (m^3^)	1.26	0.78	1.93	0.35	1.27	0.35
Emirates		Out	Electricity (kWh)	0.18	0.14	0.22	0.02	0.18	0.02
United	24,348	Out	Water (m^3^)	1.04	0.81	1.30	0.15	1.02	0.15
Kingdom		In	Electricity (kWh)	0.14	0.11	0.17	0.02	0.14	0.02
Canada	28,088	In	Water (m^3^)	0.78	0.56	1.05	0.14	0.63	0.11
		Out	Electricity (kWh)	0.11	0.09	0.14	0.02	0.09	0.02

^1^In and out of sample indicate whether or not the country was included in the data set used to estimate the model.

^2^Regional estimates are available at http://www.who.int/choice.

## Discussion

The prices of our "non-traded" intermediate inputs vary across countries with variation in GDP per capita, though not always in ways that were anticipated. Prices of printed materials and media advertising increase with GDP per capita. They increase at a slower rate than the increase in GDP per capita, except in Eastern Europe. This suggests that many of the raw materials used to produce these services are themselves traded internationally, reducing differences in the cost of production across settings. Media in Eastern Europe is an exception, where prices are lower than would be expected for the observed levels of GDP per capita, but they then increase more than proportionally to increases in national income per capita. A possible explanation is that some of the governments in the poorer ex-Soviet countries maintain tight control of media outlets which do not yet charge commercial rates for advertising, while the richer countries have been moving more towards a market-based system. Interestingly, the only domestic supply or demand variable influencing price was the size of the population served by media outlets, which seems to allow media companies to charge more because of the increased demand.

On the other hand, there is a negative relationship between the prices of water and electricity and GDP per capita. This would not be expected if market forces played the dominant role in setting prices. Demand, willingness and ability to pay would be higher in richer countries on average, so the lower prices in those settings might represent the use of more efficient, less costly technologies used to produce these services. Another possible explanation lies in the extent of government regulation or control of these markets. It might be that governments in richer countries are more willing, or more effective, in controlling prices of monopoly producers than those in poorer settings. Alternatively, where governments own the companies producing utilities, they might seek to exercise their monopoly powers to extract higher prices more in poorer than richer countries. It is not possible to verify these possible explanations with the data available to us.

In any case, the relationship with GDP per capita is mediated by local market conditions reflected in the total consumption of water and electricity. Where total consumption is higher, prices are also higher – higher demand means higher prices, and the marginal cost of production rises with increased output. It also suggests that there are no significant economies of scale which would have suggested a negative relationship with GDP per capita, other things held equal.

Overall, the models fit the data relatively well as judged by the F statistic, R squared, the tolerance test, and visual and mathematical testing for heteroskedasticity. Additionally, contacts in the countries for which out-of-sample imputation was undertaken suggested that the imputed values had face validity.

There are, of course, limitations to the analysis. For example, we were forced to take data from different countries and different years, deflate or inflate them to 2000, and analyse them as cross-sectional data. This implies that the possible impact of time-related explanatory variables is not captured. While it would have been preferable to use panel data techniques, there were insufficient observations over time and countries to allow this form of analysis. Finally, the model is not intended to predict price changes over short time intervals – such as fluctuations in the spot price of electricity – but to impute average prices over a specific time period, i.e., one year.

Policy makers seeking information on the appropriate mix of interventions necessary to achieve their health goals, or the resources required to scale up health interventions, require accurate information. This means that cost estimates should include programme costs, and that the prices used in the calculations should be representative of the range of prices likely to be found in practice. The preferred approach would be to collect a representative sample of observations for each price required for the study, specific to each country, but this is often not feasible or affordable. In this case, we have demonstrated a method in this paper to impute prices for settings where the preferred approach cannot be undertaken, that takes into account variation across and within countries. It produces price estimates that have face validity, and we hypothesize that it is a preferable alternative to basing estimates on a single price observation, or even a few observations, all of which could be outliers.

## Authors' contributions

BJ was responsible for data collection, management and analysis, participated in the development of the methodology and drafted the manuscript. TA contributed to the development of the methodology, as well as data analysis and reporting. DE took part in the development and coordination of the methodology and interpretation of the results. All authors contributed to the writing and approved the final version of the manuscript.
